# All mouth and trousers? Use of the Devil’s Advocate questioning protocol to determine authenticity of opinions about protester actions

**DOI:** 10.1080/13218719.2023.2242433

**Published:** 2023-09-19

**Authors:** Samantha Mann, Aldert Vrij, Haneen Deeb, Sharon Leal

**Affiliations:** Department of Psychology, University of Portsmouth, Portsmouth, UK

**Keywords:** arguments, clarity, devil’s advocate question, immediacy, interviewing, lie detection, opinions, plausibility, protester actions, verbal lie detection

## Abstract

We examined the Devil’s Advocate lie detection method which is aimed at detecting lying about opinions. In this approach, participants give reasons for why they hold an opinion in the eliciting-opinion question and counter-arguments to their opinion in a devil’s advocate question. Truth tellers (n = 55) reported their true opinion about protestor actions, whereas lie tellers (n = 55) reported the opposite of their true opinion. Answers were coded for number of arguments and plausibility, immediacy, clarity and scriptedness. Data were analysed with analyses of variance with veracity being the sole factor. Supporting the hypothesis, truth tellers provided more pro-arguments than lie tellers and to all eliciting-opinion questions their answers sounded more plausible, immediate and clear than lie tellers’ answers. The opposite pattern was predicted for the devil’s advocate question but not found, likely caused by the simplification of the question. Neither was being scripted a diagnostic veracity indicator.

Verbal deception research overwhelmingly focuses on lying about alleged activities (Vrij, 2008; Vrij, Fisher, et al., 2023; Vrij, Granhag, et al., 2022). Practitioners, however, frequently tell us that they are also interested in lying about opinions. Examples of these are: ‘What is this person’s view of committing terrorist acts to achieve a goal?’, ‘What does this person think of protester actions?’, ‘What is this person’s view of our Government and country?’ and ‘What is this person’s view of the network of people s/he wants to inform us about as an informant?’. Making incorrect veracity judgements about expressed opinions can have serious consequences. Usman Khan was the London Bridge attacker who stabbed two people to death and injured three others on 29 November 2019 before being shot dead by the police. He spent eight years in jail for terrorist offences but was released on parole in December 2018. He was considered a ‘success story’ for a rehabilitation programme for terrorism convicts (Dixon et al., 2019).

The Devil’s Advocate approach is to our knowledge the only verbal interview protocol available to date designed to detect lying about opinions. As far as we are aware it has been examined three times with individual interviewees (Leal, Vrij, Deeb, & Dabrowna, 2023; Leal et al., 2010; Mann et al., 2022) and once with pairs of interviewees (Deeb et al., 2018). The Devil’s Advocate approach consists of three questions. First, the interviewee is asked what his/her opinion is about a specific topic (‘What do you think of the cost-of-living protest actions that occur across the UK’?). This is followed by an eliciting-opinion question that invites interviewees to report their arguments in favour of the view they just expressed – for example, ‘Provide all the reasons why you are in favour of the cost-of-living protest actions.’ The third question is the devil’s advocate question in which interviewees are asked to give arguments opposed to their expressed view: ‘Try to play devil’s advocate and imagine that you are against the cost-of-living protest actions. Provide all the reasons why you may be against these actions’.

The Devil’s Advocate interview protocol focuses on the last two questions. Truth tellers and lie tellers are thought to respond differently to them. In this context, truth telling means expressing a true opinion whereas lying means expressing an opinion opposite to the true opinion. For example, someone who is in favour of protest actions claims in the interview to be against them. When people have formed an opinion about a topic, they have thought about reasons that support their view (Ajzen, 2001) and those reasons are typically readily available to them (Darley & Gross, 1983; Jones & Sugden, 2001). The eliciting-opinion question invites truth tellers to express these reasons, and they should be able to do so eloquently. The devil’s advocate question invites truth tellers to report reasons that oppose their actual opinion. Such reasons are less readily available to them because, as the confirmation bias predicts, people do not seek information that opposes their views (Darley & Gross, 1983; Jones & Sugden, 2001). Truth tellers should therefore struggle more to answer the devil’s advocate question than the eliciting-opinion question, resulting in a less eloquent answer to the devil’s advocate question than to the eliciting-opinion question.

For lie tellers, the devil’s advocate question is asking for the reasons they believe. These reasons should therefore be readily available to them. The eliciting-opinion question asks for reasons they do not believe and these reasons should thus be less readily available to them. Yet, for two reasons we do not predict lie tellers to be more eloquent when responding to the devil’s advocate question than to the eliciting-opinion question. First, lie tellers prepare themselves for interviews to come across as convincing (Clemens et al., 2013; Deeb et al., 2018; Leins et al., 2013). They are therefore likely to think of reasons they can give that support their pretended opinion. This should improve the eloquence of their responses to the eliciting-opinion question. Second, although truth tellers often think that their credibility shines through (Kassin, 2014; Kassin et al., 2010) – referred to as the illusion of transparency (Savitsky & Gilovich, 2003) – lie tellers do not take their credibility for granted (DePaulo et al., 2003). It means that for lie tellers the way they present information matters (Granhag & Hartwig, 2008). Lie tellers are keen to be consistent in interviews because they think that inconsistencies indicate deceit (Deeb et al., 2017; Strömwall et al., 2004; Vrij, Deeb et al., 2021). In an interview about alleged past activities, consistency is typically defined as presenting the same information in multiple interviews (Fisher et al., 2013; Vredeveldt et al., 2014). This type of consistency is impossible in a Devil’s Advocate interview because the eliciting-opinion and devil’s advocate requests ask about different things. In a Devil’s Advocate interview, consistency would mean responding to the eliciting-opinion and devil’s advocate request with equal eloquence and/or in equal length. The latter (equal length) is how consistency is defined in comparable baseline deception research (Bogaard, Meijer, et al., 2022; Bogaard, Nußbaum, et al., 2022; Palena et al., 2018), whereby a truthful answer about a certain activity is compared with the answer to a target question about which the veracity status is unknown.

The three experiments published to date supported the Devil’s Advocate reasoning. The residue means (responses to the opinion-eliciting questions minus responses to the devil’s advocate questions) were larger in truth tellers than in lie tellers for eloquence in Leal, Vrij, Deeb, and Dabrowna (2023) and Mann et al. (2022) (Leal, Vrij, Deeb, & Dabrowna, 2023: plausibility *d* = 0.51, immediacy *d* = 0.38, clarity *d* = 0.48; Mann et al., 2022: plausibility *d* = 0.47, immediacy *d* = 0.48, clarity *d* = 0.35). Leal et al. (2010) measured only plausibility and immediacy and reported their findings differently: they reported the residue means for truth tellers and lie tellers separately. The same pattern as that in Leal, Vrij, Deeb, and Dabrowna (2023) and Mann et al. (2022) emerged: in truth tellers, the residue means in truth tellers (plausibility *d* = 1.59 and immediacy *d* = 2.16) were more pronounced than in lie tellers (plausibility *d* = 0.04 and immediacy *d* = 0.36).

Unlike Leal et al. (2010), Leal, Vrij, Deeb, and Dabrowna (2023) and Mann et al. (2022) counted the number of reasons that truth tellers and lie tellers provided. For truth tellers, the arguments that support their view are more readily available than their arguments that oppose their view. For lie tellers, the pro- and anti-arguments may be more in balance due to their preparation and keenness to be consistent. It was therefore predicted that truth tellers would provide more reasons that supported their opinion (defined as pro-arguments minus anti-arguments) than lie tellers. This was not found in previous research. In fact, no difference emerged in Leal, Vrij, Deeb, and Dabrowna (2023) (*d* = 0.22), whereas lie tellers reported more pro-arguments than truth tellers in Mann et al. (2022) (*d =* 0.45). We sought to examine the robustness of these unexpected effects in the present experiment.

It has been argued that it is important to measure the strategies that truth tellers and lie tellers use in interviews to appear convincing (DePaulo et al., 2003; Vrij & Granhag, 2012). Such strategies could explain the differences in speech content (as well as nonverbal behaviour) between truth tellers and lie tellers (DePaulo et al., 2003). Insight into strategies could also be used to develop interview protocols intended to exploit the different strategies that truth tellers and lie tellers use (Vrij & Granhag, 2012; Vrij, Granhag, et al., 2022). To date, strategies have only been investigated when people tell the truth or lie about alleged activities. Results showed that truth tellers are inclined to tell it all whereas lie tellers prefer to keep their stories simple (Hartwig et al., 2007; see also Colwell et al., 2006; Hartwig et al., 2010; and Vrij et al. (2010)). Another strategy reported by both truth tellers and lie tellers is to control their nonverbal demeanour (Leal, Vrij, Deeb, & Fisher, 2023). We explored whether these and perhaps other strategies would emerge when people lie about their opinions.

Similar to Mann et al. (2022), the present experiment employed the Devil’s Advocate interview protocol in a protester-actions setting. We introduced the following six changes. First, the level of activism participants endorsed was rather low in Mann et al. (2022). We attempted to raise these levels by presenting participants with vignettes whereby a protagonist has suffered a clear injustice. The vignettes may stir up empathy/anger in participants such that they may consider it appropriate to take stronger actions about the situations described.

Second, our discussions with practitioners revealed that they found the devil’s advocate question difficult to use due to the way it was phrased. Mann et al. (2022) kept the original phrasing intact but added a question to the protocol that elicited devil’s advocate responses in a different way: participants were asked to take on the role of a script writer and to write a part in a film for a character who strongly holds the opposite view to the views they supported in the interview. It thus invited truth tellers to report views opposite to their real views but lie tellers to report their real views. This alternative devil’s advocate question did not reveal veracity differences. We therefore dropped it in the present experiment but simplified the original devil’s advocate question instead. See the Method section for the entire interview protocol including the new devil’s advocate question (Q3).

Third, there was only one eliciting-opinion question in Mann et al. (2022) and Leal et al. (2010). In the current protocol, we included three eliciting-opinion questions (Q2 is the original question, and Q4 and Q5 are new). Multiple questions about the same concept show the level of internal consistency, which is an indicator of reliability (Henson, 2001). Note that the result of the changes presented in Points 2 and 3 is that the current interview protocol is unbalanced, with three eliciting-opinion and one devil’s advocate question.

Fourth, Mann et al. (2022) examined the Verifiability approach in addition to the Devil’s Advocate approach whereas we have focused solely on the Devil’s Advocate approach. Fifth, unlike Mann et al. (2022) and Leal et al. (2010), we measured the self-reported strategies that interviewees used to come across as convincing during the interview.

Finally, there were a few deviations from Mann et al. (2022) regarding these dependent variables. We did not measure *number of words*, *number of details* and *verifiable sources*. Number of words and number of details were dropped because they did not yield significant effects in Mann et al. (2022). In addition, we considered number of words to be too vague to be meaningful. Available methods to code details typically refer to references to people, location, actions, time and objects (Alison et al., 2013; Deeb et al., 2022) or to references to sensory information (details related to vision, sound, taste, touch or smell) and contextual embeddings (where and when an experience took place; Gancedo et al., 2021). Such details in coding makes sense when people discuss alleged experiences but makes less sense when people discuss their opinions. Verifiable sources were deleted because they are part of the Verifiability approach rather than the Devil’s Advocate approach.

The variable *being scripted* is new. Scriptedness is defined as giving a response that contains mental scripts – for example, two- or three- or more-word phrases that automatically come to mind in speech (e.g. ‘to be honest’, ‘knock-on-effect’, ‘social media platform’, etc.). All other variables we measured are cues to truthfulness (truth tellers report them more than lie tellers), whereas being scripted would be a cue to deceit (lie tellers report it more than truth tellers). There is a lack of cues to deceit in verbal lie detection (Nahari et al., 2019), which is considered problematic (Vrij, Fisher, et al., 2023). If only cues to truthfulness are measured, deception can only be determined indirectly: via the absence of cues to truthfulness. In real life someone could be more confident in deciding that someone is lying if the absence of cues to truthfulness is accompanied by the presence of cues to deceit (Vrij, Fisher, et al., 2023).

## Hypothesis

In this pre-registered experiment (pre-registration: https://osf.io/f5chx?view_only=509da9433071416490e8461a12e7c995) we tested one pre-registered hypothesis. It was similar to the hypothesis that was examined by Mann et al. (2022). Truth tellers will provide more arguments defending their opinion, and their responses will be more plausible, immediate and clear than those of lie tellers. Lie tellers’ responses will sound more 'scripted’.

A more direct test of the Devil’s Advocate Approach is to compare the answers to the eliciting-opinion question and the devil’s advocate questions. For this purpose, we subtracted the devil’s advocate mean score from the eliciting-opinion mean score, and, following Mann et al. (2022), we label these mean scores ‘residue means’. The Devil’s Advocate approach predicts higher residue means for truth tellers than for lie tellers (exploratory hypothesis).

## Method

### Participants

We ran a GPower analysis to determine the required sample size for a multivariate *F*-test (multivariate analysis of variance, MANOVA, global effects). Assuming an alpha level of .05, a statistical power of .85, and a medium effect size (*f*^2^ = .16), we needed at least 102 participants for the experiment. We aimed for a medium effect size as our smallest effect size of interest due to the practical significance of the research. Differences between truth tellers and lie tellers should be clearly noticeable in real time, a prerequisite for making a veracity assessment method relevant for practitioners. This smallest effect size of interest also aligns with previous research on the Devil’s Advocate approach (e.g. Mann et al., 2022) that showed medium to large effect sizes. We recruited 112 adult participants, but two participants did not follow the instructions correctly. Their data were omitted, and the final sample included 110 participants. Most participants were females (70%); the others were males (28.2%) or non-binary (1.8%). Their mean age was 28.86 years (*SD* = 11.25). Most participants (64.5%) were from the United Kingdom; the others were from Eastern Europe (10.9%), Western Asia (9.1%), Western Europe (6.4%), Africa (4.5%), Eastern Asia (1.8%) or the Middle East (0.9%). Participants were recruited via the departmental database and university staff and student portals; 49.1% of participants were students at the university, 18.2% were staff, and 32.7% were not from the university. A total of 18 different native languages were spoken across the sample, but for most participants (70%) English was the native language. The experiment conformed with the principles of the Declaration of Helsinki, and ethics approval was granted by the University faculty’s ethics committee and the sponsor’s ethics committee.

### Statistical analysis

We carried out the same analyses as those of Mann et al. (2022). To test the pre-registered hypothesis, we carried out MANOVAs with veracity as the only factor and as dependent variables the number of pro- and anti-arguments and (via 7-point Likert scales) ratings for plausibility, immediacy, clarity and being scripted. Pro-arguments are arguments supporting the views expressed by the interviewee: arguments that could be expected when expressing the attitude and when answering the eliciting-opinion question. Anti-arguments are arguments opposing the views expressed by the interviewee: arguments that could be expected when answering the devil’s advocate question.

A direct test of the Devil’s Advocate approach is to compare the answers to the eliciting-opinion questions (Q2, Q4 and Q5) and the devil’s advocate question (Q3). Thus, in the second MANOVA we analysed the difference in answering the three eliciting opinion questions (by averaging the results to the three individual questions) and the devil’s advocate question. Following Mann et al. (2022), we subtracted the devil’s advocate mean score from the eliciting-opinion mean score (e.g. average plausibility score in the three eliciting opinion questions minus the plausibility score in the devil’s advocate question). The pro-action arguments residue mean was computed as: (eliciting-opinion questions pro-action arguments + devil’s advocate question pro-action arguments) – (eliciting-opinion questions anti-action arguments + devil’s advocate question anti-action arguments). Following Mann et al. (2022), we label these mean scores ‘residue means’.

Note that the pre-registered hypothesis and exploratory hypothesis refer to the same data. The difference is that they are analysed in different ways. The pre-registered hypothesis analyses present a complete picture of the results. The exploratory hypothesis is based on residue means. It masks the individual eliciting-opinion and devil’s advocate results but is a more direct test of the Devil’s Advocate approach. In other words, the two different sets of analyses have different strengths, which justifies presenting both.

### Procedure

Participants were recruited via an advertisement entitled ‘Actions speak louder than words: opinions about protester actions’ and which mentioned that participants in the experiment would be asked to either lie or tell the truth regarding their beliefs about activist behaviour.

The experiment took place online via Zoom. Those who expressed an interest to take part were sent a Qualtrics link to the Participant Information Sheet (PIS) that outlined the experiment and the consent form. Participants were asked to read the PIS form and to sign their consent if they agreed to take part. After receiving the signed informed consent form and prior to the experiment taking place, the experimenter sent the participant a Qualtrics link to the first questionnaire. It comprised a request for usual demographic information (gender, age, nationality, first language, etc.) followed by four vignettes (each relating to a different topic) depicting a scenario whereby a protagonist has suffered an injustice. The vignettes were designed to stir up empathy/anger in participants such that they may consider it appropriate for action to be taken about the situations described. The order in which the vignettes were presented was alternated to avoid any fatigue effects. See Appendix for the four vignettes.

The participant was asked to indicate on a 10-point scale how unfair the situation described in the vignette was (from 1 = *low* to 10 = *high*) and the extent to which they sympathise with the protagonist in the vignette (from 1 = *not at all* to 10 = *very much so*). They were then asked to rank the four vignettes in order of their perceived level of unfairness.

Once the experimenter received responses to this questionnaire she sent the participant a link to the second questionnaire, which asked 48 questions relating to what actions participants would consider to be appropriate for the protagonist to take in the example given in the vignette that they ranked as most unjust. The aim of the questionnaire was to establish ground truth of actions the participant considers appropriate for someone else to take. The questionnaire is based on the Activism Orientation Scale (Corning & Myers, 2002) and was also used by Mann et al. (2022). Unbeknown to the participant, the items in the questionnaire distinguish between six levels of activism, whereby Level 1 indicates that the participant condones only low levels of protest (e.g. displaying slogan stickers), and Level 6 indicates condoning high levels of protest (e.g. using physical violence). See Table 1 for the six levels and an example of each level. As in the Activism Orientation Scale, the participant was asked to rate their agreement to each item on a 4-point Likert scale (completely disagree/somewhat disagree/somewhat agree/completely agree).

**Table 1.** The six levels of activism and an example of each level.

Order level	Example
1. Public protest/passive support for cause	Display a poster or car sticker with a political message
2. Civil disruption/slightly active (lawful) support for cause	Attend an informational meeting of a relevant political group
3. Social disruption/persuade or try to convince others of view and to support cause	Invite a friend to attend a meeting of a relevant political organisation or event
4. Civil disobedience	Use tactics designed to overwhelm police resources to make their point
5. Criminal damage	Graffiti an opinion on public property to make their point
6. More extreme criminal actions/risk personal safety	Use physical violence to make their point

At the agreed time for the experiment to take place, the experimenter met with the participant and briefly explained that they would be interviewed shortly about the vignette and the actions that they considered appropriate for the protagonists to take. She then posted the vignette that the participant had previously selected as the most unjust into the chat feature of Zoom and asked the participant to read it again prior to being interviewed. The experimenter then briefly discussed with the participant their responses to the second questionnaire. She then informed the participant either that they are to answer completely honestly in their interview (truth tellers, *n* = 55) or to tell the interviewer that they would only condone a much lower level of action (or no action) than they consider to be appropriate (lie tellers, *n* = 55). To increase motivation, the participant was told that in addition to their £10 for taking part, if they successfully convince the interviewer that they are being truthful they will be entered into a draw for £50 Amazon vouchers, but if they are not successful, they will be asked to write a statement about their real beliefs. In fact, all participants were entered into the draw (and not asked to write a statement). The participant was then given as much time as they wanted if they wanted to prepare a rationale for their beliefs in response to possible questions (more relevant for lie tellers who were giving responses that do not reflect their actual beliefs).

When the participant indicated that they were ready to be interviewed, they were put into a break-out room on Zoom with the interviewer who asked the following five questions:

Q1. What actions do you think it would be appropriate for Jane (and Matt) to take in their situation, to express how unfairly Jane has been treated? (*Expressing actions question*)

Q2. What has led you to feel that this action [just described in 1] is appropriate? (*First eliciting-opinion question*)

Q3. Can you think of any arguments for why Jane and Matt should not take those actions against those individuals? (*Devil’s advocate question*)

Q4. Why might someone decide to act in this situation? (*Second eliciting-opinion question*)

Q5. Why do you think that stronger actions than those you discussed earlier would be inappropriate? (*Third eliciting-opinion question*)

The interviewer notified the experimenter when the interview had concluded, after which the experimenter brought the participant back into the main meeting room. Participants were asked to complete a post-interview questionnaire where they rated on a percentage scale the extent to which they had told the truth. They were then debriefed. All participants received £10 (via a bank transfer) and were entered in the £50 Amazon vouchers draw.

### Coding

All interviews were recorded. An audio file was retained at the end of the interview for transcribing. All interviews were transcribed and coded for each question. One rater, blind to the veracity status of the participant, coded the number of pro-arguments, number of anti-arguments and, on 7-point Likert scales, plausibility, immediacy, clarity and being scripted.

In the Devil’s Advocate protocol each question is considered an individual entity. Therefore, when repetitions occurred within the same question (e.g. in Q1) the information was not coded again, but if they occurred in another question they were coded again (e.g. in Q1 and in Q2). We coded the number of pro-arguments, number of anti-arguments and, on 7-point Likert scales, plausibility, immediacy, clarity and being scripted. The way Questions 2 and 4 (both eliciting-opinion questions) were phrased implies that they may result in participants repeating their Question 2 answer in Question 4. This did not happen frequently. Only five truth tellers and four lie tellers repeated a pro-argument raised in Question 2 in Question 4 and only three participants (all lie tellers) repeated an anti-argument mentioned in Question 2 in Question 4. Percentage-wise, 2% (*SD* = 8%) of the pro-arguments and 1% (*SD* = 7%) of the anti-arguments were repeated in Question 4, with no significant difference in repetitions between truth tellers and lie tellers for pro-arguments, *F*(1, 108) = 0.66, *p* = .418, *d* = 0.12, 95% confidence interval, CI [–0.25, 0.50], and anti-arguments, *F*(1, 108) = 3.02, *p* = .085, *d* = 0.28, 95% CI [–0.10, 0.65]. We decided to keep the repeated arguments in the dataset.

For Question 1 we coded pro-action and anti-action arguments that the participants mentioned. For example, this statement contained three pro-action arguments (in bold) and one anti-action argument (in italics).

So I think it’ll be suitable, let’s say acceptable for them to um **organise uh Facebook group** or some sort of **campaign** and advertise it online to get other people who have experienced the same thing and um share their beliefs or their the issues they’ve had just to kind of create a bigger group and express the issues they’ve had to then uh political party or um **start a petition,** maybe and just speak to someone who is in charge, and is able to actually influence the change they want. Um so more of um what’s the word lenient approach I would say, just because it’s not, I don’t think it’s acceptable for anyone to be, you know, *uh violent, uh express their opinions, like, throw eggs at the parliament*, something, I wouldn’t say that that’s appropriate.

For Questions 2 to 5 we coded the number of reasons participants came up with in favour of (pro-arguments) or against (anti-arguments) the appropriateness of the actions they described in their answers to Question 1. For example, this statement contained two pro-arguments (in bold) and two anti-arguments (in italics).

If you I believe in protest, **I believe in protest in the most ethical remit**. *I don’t believe in obstructing other people’s day.* Because I think actually you can end up *inadvertently hurting people who probably agree with your cause*, just because the avenue you went down to express yourself. So it also it’s getting your voice **heard in the right forums, even if those forums aren’t necessarily gonna bring an immediate benefit, you lend your voice to a cause.** And I think that’s the best way to bring about change, rather than drawing a lot of attention to yourself in the wrong format, if that makes sense.

Questions 1 to 5 were also rated on scales of 1 (not at all) to 7 (extremely) for plausibility (whether the response sounds reasonable and genuine and if there is enough of an answer to sound convincing), immediacy (whether the response sounds personal and not distanced), clarity (how clear the response is for the reader to understand what the participant is saying by the end of the answer) and being scripted (a response that contains mental scripts, e.g. two-, three- or more-word phrases that automatically come to mind in speech). The definitions for plausibility, immediacy and clarity were taken from Mann et al. (2022) but being scripted was a new variable not coded by Mann et al. (2022). The following example received a 6 for plausibility, immediacy and directness and a 2 for being scripted:

uh so I think, **participating in political meetings** and **sharing online posts, those are okay, politically**, cause **you’re not being a threat to anyone in society**, you’re not endangering anyone, you’re not **getting physically violent**, or uh **obstructing other people in their day** to day lives.

A ‘6′ for plausibility was given because the answer was thoughtfully and carefully worded. Immediacy received a ‘6’ because it was to the point and personal, insofar as the participant gives an opinion that starts with ‘I think’ and lists what they think is acceptable or not acceptable. Clarity received a ‘6’ because the participant put effort into explaining their views, and the answer was well understood by the coders. Being scripted received a ‘2’ because the answer included phrases such as ‘physically violent’, ‘online posts’ and ‘day to day lives’.

The following answer received a ‘2’ for plausibility, immediacy and clarity and a ‘1’ for being scripted, because the person struggles to come up with a meaningful answer.

well I guess it’s a personal thing, isn’t it? So for the, for the for the person, the person has been working hard to get to that place. Um and now they feel that they’ve been they’ve been sort of, yeah, let down by the university, or, you know but yeah but yeah.

A second rater, also blind to the veracity status of the participants, coded the interviews of a random sample of 54 participants (49%). Inter-rater reliability between the two coders was measured using the two-way random effects model measuring consistency. It was good to very good for pro-action arguments (average measures intraclass correlation coefficient, ICC = .98), anti-action arguments (average measures ICC = .94), plausibility (average measures ICC = .75), immediacy (average measures ICC = .86) and clarity (average measures ICC = .81) and satisfactory for being scripted (average measures ICC = .61; Table 2).

**Table 2.** Level of action allocation as a function of veracity.

Level	Truth tellers		Lie tellers	
	*N*	%	*N*	%
1	27	49.1	25	45.5
2	3	5.5	3	5.5
3	23	41.8	23	41.8
4	1	1.8	1	1.8
5	1	1.8	0	0
6	0	0	0	0

For coding the self-reported strategies, a bottom–up form of coding was carried out. One rater grouped similar comments together and categorised them accordingly. This resulted in the 10 categories listed in Table 3. A second rater then allocated each comment in one or more of the 10 categories. The inter-rater reliability between the two coders was very good, kappa = .82. The discrepancies between the two coders were resolved by a third coder.

**Table 3.** The self-reported strategies used by lie tellers and truth tellers.

Strategy	Example	Lie tellers	Truth tellers
Improvise	Go with the flow/thought quickly	8	0
Speech-related	Repeating questions, pretend not to be knowledgeable, used a conversational style	7	0
Nonverbal demeanour	Controlling behaviour such as hands, eye contact or appearing confident to interviewer	5	1
Truth	Based answer on their own opinions etc	4	6
Reverse	Thought of own opinion and said the opposite	4	0
Consistency	Consistent answers	4	0
Example	Based answer on an example and kept building on that	2	3
Solution	Came up with a specific solution to problem e.g. ‘I said they should write to the university’	1	2
Other person perspective	Thought of someone with opposing views and imagined what they would say	1	1
Facts	Based answer on facts they know/other people’s anecdotes etc.	0	3

## Results

### Manipulation check: levels of unfairness, sympathy and appropriate actions

Two analyses of variance (ANOVAs) were carried out with veracity as the only factor and the perceived levels of unfairness and sympathy related to the vignette they discussed in the interview as dependent variables. The effects for unfairness, *F*(1, 108) = 0.00, *p* = 1.00, *d* = 0.00, 95% CI [–0.37, 0.37] and sympathy were not significant, *F*(1, 108) = 0.73, *p* = .396, *d =* 0.17, 95% CI [–0.21, 0.54]. The total means showed that the participants found the situation described in the vignette very unfair (*M* = 8.96, *SD* = 1.26), and they felt a lot of empathy for the protagonists described in the vignette (*M* = 8.91, *SD* = 1.57). The chosen topics for truth tellers to discuss in the interview were cannabis (*n* = 14, 25.5%), university fees (*n* = 12, 21.8%), cancer/Covid (*n* = 12, 21.8%) and cost of living crisis (*n* = 17, 30.9%). The topics chosen for lie tellers were cannabis (*n* = 11, 20.0%), university fees (*n* = 11, 20.0%), cancer/Covid (*n* = 18, 32.7%) and cost of living crisis (*n* = 15, 27.3%). There was no association between veracity and the chosen vignette, χ^2^(3, *N* = 110) = 1.73, *p* = .631, θ = .13.

A chi-square test of independence was performed to examine the relationship between veracity and the highest level of actions considered appropriate by the participants. There was no association between these two variables, χ^2^(4, *N* = 110) = 1.26, *p* = .868, θ = .11. See Table 2 for the chosen levels, which were generally low.

### Manipulation check: reported truth telling

An ANOVA with veracity (truth vs. lie) as the only factor and the percentage of reporting truth telling as the dependent variable revealed a significant effect, *F*(1, 108) = 336.44, *p* < .001, *d =* 3.49, 95% CI [2.85, 4.03]. Truth tellers (*M* = 97.33%, *SD* = 5.52, 95% CI [92.16, 102.49]) reported to have told the truth more than lie tellers (*M* = 29.73%, *SD* = 26.80, 95% CI [24.56, 34.89]).

### Reported strategies

More lie tellers (*n* = 25, 45.5%) than truth tellers (*n* = 13, 23.6%) reported to have used a strategy in the interview to appear convincing, χ^2^(2, *N* = 110) = 5.79, *p* = .016, θ = .34. The reported strategies are depicted in Table 3. The most frequently reported strategy amongst lie tellers (*n* = 8) was improvisation followed by a speech-related strategy that was not speech-content related (repeating questions, pretending lack of knowledge, use a conversational style) followed by paying attention to nonverbal behaviour. The most frequently reported strategy amongst truth tellers was telling the truth.

### Pre-registered hypothesis testing

We tested our data using null hypothesis significance testing (NHST) and equivalence testing to support any null findings demonstrating an absence of differences between truth tellers and lie tellers (see Lakens et al., 2018). We chose our smallest effect size of interest to be 0.5, because our research is applied, and we were interested in observing a moderate to large effect size. Thus, the equivalence bounds ranged between −0.5 and 0.5.

A MANOVA analysing the expressing attitude question (Q1) with veracity as the only factor and the six variables listed in Table 4 as dependent variables revealed a significant multivariate veracity effect, *F*(6, 103) = 22.52, *p* < .001, $\eta_{p}2$ = .57. Table 4 shows that our hypothesis was supported for all the dependent variables except for being scripted, for which the null hypothesis was supported. Truth tellers reported more pro- and fewer anti-arguments than lie tellers about the actions that would be appropriate to take. Compared to lie tellers’ statements, the truth tellers’ statements were rated as more plausible, immediate and clear.

**Table 4.** Statistical results for the expressing actions question, eliciting-opinion questions and devil’s advocate question.

	Truth			Lie			NHST			Equivalence testing	
	*M*	*SD*	95% CI	*M*	*SD*	95% CI	*F*	*p*	*d* [95% CI]	*t*	*p*
**Expressing actions question (Q1)**											
Pro-action arguments	2.73	1.87	2.36, 3.10	0.29	0.57	−0.08, 0.66	85.48	<.001	1.77 [1.30, 2.18]	7.35	1.00
Anti-action arguments	0.45	0.77	0.16, 0.75	0.87	1.36	0.58, 1.17	3.94	.050	0.38 [0.00, 0.75]	0.39	.349
Plausibility	4.71	0.94	4.49, 4.93	3.20	0.68	2.98, 3.42	93.75	<.001	1.84 [1.37, 2.26]	6.47	1.00
Immediacy	4.33	0.90	4.12, 4.54	3.29	0.66	3.08, 3.50	47.29	<.001	1.32 [0.89, 1.71]	3.56	1.00
Clarity	4.38	0.99	4.14, 4.63	3.64	0.82	3.39, 3.88	18.40	<.001	0.81 [0.41, 1.19]	1.41	.920
Being scripted	2.18	0.67	1.98, 2.38	1.98	0.80	1.78, 2.18	2.01	.159	0.27 [–0.11, 0.64]	−2.13	.018
**Three eliciting-opinion** **questions** **(** **averaged)**											
Pro-action arguments	3.02	2.63	2.45, 3.59	1.02	1.44	0.45, 1.59	24.35	<.001	0.94 [0.54, 1.32]	3.70	1.00
Anti-action arguments	3.16	1.90	2.54, 3.79	3.64	2.72	3.01, 4.26	1.11	.294	0.20 [–0.17, 0.58]	0.06	.476
Plausibility	4.39	0.88	4.19, 4.58	3.19	0.54	2.99, 3.38	74.44	<.001	1.64 [1.19, 2.05]	5.03	1.00
Immediacy	3.98	0.85	3.78, 4.18	3.15	0.63	2.95, 3.35	33.18	<.001	1.11 [0.69, 1.49]	2.27	.987
Clarity	4.17	0.92	3.95, 4.39	3.18	0.68	2.96, 3.39	41.30	<.001	1.22 [0.80, 1.61]	3.19	.999
Being scripted	1.95	0.61	1.78, 2.12	1.82	0.65	1.65, 1.99	1.22	.272	0.21 [–0.17, 0.58]	−3.03	.002
**Devil’s advocate** **question**											
Pro-reason arguments	0.64	0.78	0.47, 0.80	0.16	0.37	0.00, 0.33	16.49	<.001	0.79 [0.39, 1.16]	−0.23	.408
Anti-reason arguments	0.89	0.99	0.61, 1.17	0.76	1.05	0.49, 1.04	0.43	.516	0.13 [–0.25, 0.50]	−1.91	.029
Plausibility	4.45	0.98	4.22, 4.69	3.15	0.73	2.92, 3.38	63.25	<.001	1.50 [1.06, 1.90]	4.92	1.00
Immediacy	4.05	0.95	3.83, 4.28	3.05	0.73	2.83, 3.28	38.24	<.001	1.18 [0.76, 1.57]	3.09	.999
Clarity	4.22	1.10	3.95, 4.49	3.22	0.90	2.95, 3.49	27.31	<.001	1.00 [0.58, 1.38]	2.61	.995
Being scripted	1.96	0.86	1.74, 2.18	1.60	0.78	1.38, 1.82	5.37	.022	0.44 [0.05, 0.81]	−0.87	.193

Note: NHST = Null-hypothesis significance testing; CI = confidence interval.

Table 4 further shows the results for the answers to the averaged eliciting-opinion questions and devil’s advocate question separately. The MANOVA for the averaged eliciting-opinion questions showed a significant multivariate effect, *F*(6, 103) = 19.47, *p* < .001, $\eta_{p}2$ = .53. All effects except those for number of anti-arguments (for which the results were inconclusive) and being scripted (for which the null hypothesis was supported) were statistically different but not equivalent. In alignment with the Devil’s Advocate approach rationale and supporting our hypothesis, truth tellers reported more pro-arguments than lie tellers, and truth tellers’ statements appeared more plausible, immediate and clear than lie tellers’ statements.

The MANOVA for the devil’s advocate question also showed a significant multi-variate effect, *F*(6, 103) = 11.97, *p* < .001, $\eta_{p}2$ = .41. All univariate effects were statistically different and not equivalent, except the effect for number of anti-arguments reported for which the null hypothesis was supported. Table 4 shows that truth tellers reported more pro-arguments than lie tellers, and truth tellers’ statements appeared more plausible, immediate, clear and being scripted. These findings contradict the Devil’s Advocate approach rationale.

Since the devil’s advocate results were unexpected, we carried out further analyses. We examined whether the unexpected results were caused by participants avoiding answering the question by including phrases such as ‘no’ or ‘not really’ in their replies. More truth tellers (*n* = 18) than lie tellers (*n* = 8) did this, χ^2^(2, *N* = 110) = 5.04, *p* = .025. In addition, compared to the other answers, these ‘I cannot’ answers were rated as more plausible (*M* = 4.19, *SD* = 0.89, 95% CI [3.78, 4.61] vs. *M* = 3.68, *SD* = 1.11, 95% CI [3.45, 3.91]), *F*(1, 108) = 4.63, *p* = .034, *d* = 0.51, 95% CI [0.12, 0.88], and clearer (*M* = 4.15, *SD* = 1.01, 95% CI [3.73, 4.58], vs. *M* = 3.58, *SD* = 1.12, 95% CI [3.35, 3.82]), *F*(1, 108) = 5.38, *p* = .022, *d* = 0.53, 95% CI [0.15, 0.91], and more scripted (*M* = 2.08, *SD* = 0.98, 95% CI [1.76, 2.40] vs. *M* = 1.69, *SD* = 0.78, 95% CI [1.51, 1.87]), *F*(1, 108) = 4.34, *p* = .040, *d* = 0.44, 95% CI [0.06, 0.81]).

Table 5 shows the statistical results for each of the three eliciting-opinion questions. The findings for each question were similar and showed similar patterns as those that emerged in the averaged eliciting-opinion question results.

**Table 5.** Statistical results for the three individual eliciting-opinion questions.

	Truth			Lie			NHST			Equivalence testing	
	*M*	*SD*	95% CI	*M*	*SD*	95% CI	*F*	*p*	*d* [95% CI]	*t*	*p*
**Q2. Multivariate *F*(6, 103) = 11.14, *p*< .001,** $\eta_{p}2$ *=* **.39**											
Pro-action arguments	1.64	1.79	1.27, 2.01	0.42	0.79	0.05, 0.79	21.38	<.001	0.88 [0.48, 1.26]	2.73	.996
Anti-action arguments	0.55	1.02	0.24, 0.85	0.69	1.25	0.39, 1.00	0.45	.503	0.12 [–0.25, 0.49]	1.64	.052
Plausibility	4.53	0.94	4.30, 4.76	3.18	0.80	2.95, 3.42	65.65	<.001	1.55 [1.10, 1.95]	5.09	1.00
Immediacy	4.09	1.01	3.85, 4.34	3.16	0.81	2.92, 3.41	28.35	<.001	1.02 [0.60, 1.40]	2.45	.992
Clarity	4.31	1.09	4.04, 4.58	3.35	0.91	3.08, 3.61	25.49	<.001	0.96 [0.55, 1.34]	2.43	.992
Being scripted	1.95	0.80	1.72, 2.17	1.76	0.88	1.54, 1.99	1.28	.261	0.23 [–0.15, 0.60]	−1.98	.025
**Q4. Multivariate *F*(6, 103** **]** **= 9.43, *p*< .001,** $\eta_{p}2$ *=* **.36**											
Pro-action arguments	0.95	0.89	0.74, 1.16	0.42	0.66	0.21, 0.63	12.47	<.001	0.68 [0.28, 1.05]	0.18	.572
Anti-action arguments	0.11	0.37	−0.10, 0.32	0.45	1.03	0.25, 0.66	5.45	.021	0.44 [0.05, 0.81]	1.04	.150
Plausibility	4.27	0.99	4.05, 4.49	3.24	0.61	3.02, 3.46	43.80	<.001	1.25 [0.83, 1.64]	3.43	1.00
Immediacy	3.89	0.92	3.67, 4.12	3.11	0.76	2.88, 3.33	23.67	<.001	0.92 [0.52, 1.30]	1.75	.959
Clarity	4.13	1.02	3.88, 4.37	3.09	0.80	2.85, 3.33	35.19	<.001	1.13 [0.72, 1.52]	3.07	.999
Being scripted	1.93	0.72	1.73, 2.12	1.71	0.74	1.52, 1.90	2.48	.118	0.30 [–0.08, 0.67]	−2.03	.022
**Q5. Multivariate *F*(6, 103) = 13.71, *p*< .001,** $\eta_{p}2$ *=* **.44**											
Pro-action arguments	0.44	0.88	0.24, 0.63	0.18	0.55	−0.01, 0.38	3.35	.071	0.35 [–0.03, 0.73]	−1.76	.041
Ant-action arguments	2.50	1.65	2.05, 2.97	2.49	1.78	2.03, 2.95	0.003	.956	0.01 [–0.37, 0.38]	−1.47	.072
Plausibility	4.36	1.08	4.13, 4.60	3.15	0.62	2.91, 3.38	52.74	<.001	1.37 [0.94, 1.77]	4.28	1.00
Immediacy	3.95	0.99	3.71, 4.18	3.18	0.77	2.95, 3.42	20.37	<.001	0.87 [0.46, 1.25]	1.56	.939
Clarity	4.07	1.14	3.82, 4.33	3.09	0.75	2.83, 3.35	28.56	<.001	1.02 [0.60, 1.40]	2.62	.995
Being scripted	1.98	0.80	1.76, 2.21	1.98	0.87	1.76, 2.21	0.00	1.00	0.00 [–0.37, 0.37]	3.13	.001

Note: NHST = null-hypothesis significance testing.

### Exploratory hypothesis testing

The MANOVA for the residue means (means for the three eliciting-opinion questions, averaged, minus means for the devil’s advocate question) showed a significant multivariate veracity effect, *F*(5, 104) = 6.69, *p* < .001, $\eta_{p}2$ = .24. At a univariate level only the effects for number of pro-arguments supported our hypothesis, see Table 6. The residue mean was significantly higher for truth tellers than for lie tellers. For all other variables, there was statistical equivalence, meaning that the null hypothesis was supported. This supports the hypothesis for number of arguments only.

**Table 6.** Statistical results for the residue scores.

	Truth			Lie			NHST			Equivalence testing	
	*M*	*SD*	95% CI	*M*	*SD*	95% CI	*F*	*p*	*d* [95% CI]	*t*	*p*
Pro-reason arguments	−0.40	2.51	−1.16, 0.36	−3.22	3.15	−3.98, –2.46	26.91	<.001	0.99 [0.58, 1.37]	4.27	1.00
Plausibility	−0.07	0.68	−0.23, 0.10	0.04	0.53	−0.12, 0.21	0.88	.351	0.18 [–0.20, 0.55]	3.35	<.001
Immediacy	−0.08	0.68	−0.26, 0.11	0.10	0.70	−0.09, .028	1.79	.184	0.26 [–0.12, 0.63]	2.47	.008
Clarity	−0.05	0.87	−0.26, 0.16	−0.04	0.66	−0.25, 0.17	0.002	.967	0.01 [–0.36, 0.39]	3.34	<.001
Being scripted	−0.01	0.77	−0.21, 0.19	0.22	0.71	0.02, 0.42	2.65	.106	0.31 [–0.07, 0.68]	1.91	.03

Note: NHST = Null-hypothesis significance testing.

Following Mann et al. (2022), we further examined whether the residue means differed from zero for truth tellers and lie tellers. One-sample *t*-tests showed that for truth tellers none of the residue scores differed from zero in the NHST analysis (all *t*s < 1.19, all *p*s > .243, all *ds* < 0.16). In the equivalence testing analysis, all residue scores were statistically equivalent (3.84 ≤ *t*s ≤ 4.73, all *p*s < .001, −0.12 < *d*s < −0.02) except for pro-arguments, *t* = 0.30, *p* = .385, *d* = −0.16, 95% CI [–0.39, 0.06]. This confirms that all residue scores did not differ from zero; however, the results were inconclusive for pro-arguments.

For lie tellers, the residue scores for pro-arguments, *t*(54) = 7.58, *p* < .001, *d* = 1.02, 95% CI [1.02, 1.35]), and being scripted, *t*(54) = 2.26, *p* = .028, *d =* 0.31, 95% CI [0.03, 0.57]), differed from zero in the NHST analysis (all other *t*s < 1.04, all *p*s > .305, all *d*s < 0.14). These results were supported in the equivalence testing analysis for pro-arguments, *t* = −6.40, *p* = 1.00, *d* = −1.02, 95% CI [−1.33, −0.77], for plausibility, immediacy and clarity (5.11 ≤ *t*s ≤ 7.55, all *ps* < .001, −0.06 < *d*s < 0.14), and for being scripted, *t* = −2.92, *p* = .003, *d* = 0.31, 95% CI [0.08, 0.55].

## Discussion

We predicted in the pre-registered hypothesis that truth tellers’ responses to the expression opinion question (Q1) and eliciting opinion questions (Q2, Q4, Q5) would be more plausible, immediate and clear than lie tellers’ responses. We found strong support for this. It replicated Leal et al. (2010), Leal, Vrij, Deeb, and Dabrowna (2023) and Mann et al. (2022) and the findings of an experiment outside a Devil’s Advocate context where participants lied about opinions (Vrij, Deeb, et al., 2022). It strengthens the conclusion that lying about opinions can be detected by examining these three variables.

We further predicted in the pre-registered hypothesis that truth tellers would provide more arguments defending their opinion than lie tellers when answering Q1, Q2, Q4 and Q5. We found strong support for this hypothesis for the number of pro-arguments mentioned (truth tellers reported more pro-arguments than lie tellers). This contradicts Mann et al. (2022) who found that lie tellers reported more pro-arguments than truth tellers. Leal, Vrij, Deeb, and Dabrowna (2023) and Vrij, Deeb, et al. (2022) found no significant veracity effect for the number of pro-arguments. In other words, the four available experiments to date showed all three possible effects. Compared to lie tellers, truth tellers reported (a) more, (b) fewer or (c) the same number of pro-arguments. Although more research into this variable is required to determine whether it is a diagnostic veracity indicator when people lie about their opinions, its potential to become a diagnostic indicator looks bleak.

There was weak support for the pre-registered hypothesis that lie tellers report more anti-arguments than truth tellers. Limited support was also found by Vrij, Deeb, et al. (2022) but Leal, Vrij, Deeb, and Dabrowna (2023) and Mann et al. (2022) found no veracity effect for this variable. Again, more research is required to determine the diagnostic value of this variable as a veracity indicator when people lie about their opinions.

The results contradicted the predictions for the devil’s advocate question (Q3) because truth tellers sounded more (rather than less) plausible, immediate and clear than lie tellers when answering the devil’s advocate question. The consequence of the unexpected effect for the devil’s advocate question is that the exploratory hypothesis largely failed to show the expected results, unlike Leal et al. (2010), Leal, Vrij, Deeb, and Dabrowna (2023) and Mann et al. (2022), where the devil’s advocate question yielded the expected results. The exploratory hypothesis predicted higher residue means for truth tellers than for lie tellers. Only the effect for number of pro-arguments supported our exploratory hypothesis: the residue mean was significantly higher for truth tellers than for lie tellers. No significant effects emerged for the remaining variables.

Being scripted, a new variable in the Devil’s Advocate approach, did not emerge as a diagnostic veracity indicator in the experiment. The ICC for this variable was lower than that for the other variables indicating that coders found it relatively difficult to code this variable. In other words, our operationalisation of being scripted was unsuccessful.

Strategies that truth tellers and lie tellers reported to have used to appear convincing in the interview were measured for the first time in a lying about opinions setting. Only a minority of lie tellers (45.5%) and a minority of truth tellers (23.6%) reported to have used a strategy. Although our dependent variables were all related to speech-content, many strategies that lie tellers reported to have used were hardly or not at all related to speech content. That is, the most frequently mentioned strategy amongst lie tellers – improvisation – is speech-content related but cannot be considered a strong strategy. This was followed by using a speech-related strategy that is not speech-content related (repeating a question, pretending not to be knowledgeable or using a conversational style) followed by paying attention to nonverbal behaviour. Lie tellers reported several speech-content related strategies, such as (a) basing their answers around the truth, (b) building their argument around an example, (c) providing the opposite of their own view, (d) consistency, (e) coming up with a solution to the protagonists’ problem and (f) using another person perspective. However, these strategies were only occasionally mentioned (these six strategies combined were mentioned 16 times). The absence of speech-content-related strategies amongst lie tellers could perhaps explain why we obtained such large veracity effects in the current experiment.

The most frequently reported strategies to be convincing when people tell the truth or lie about their alleged activities are ‘telling it all’ (truth tellers), ‘keeping the story simple’ (lie tellers) and ‘controlling nonverbal demeanour’ (lie tellers; Hartwig et al., 2007; Leal, Vrij, Deeb, & Fisher, 2023). Of these three strategies, only the controlling nonverbal demeanour strategy emerged in the present experiment. This suggests that interviewees use other strategies when they attempt to be convincing in opinion interviews rather than in interviews about alleged activities. This makes it worthwhile to further examine strategies used in opinion interviews.

The dependent variables plausibility, immediacy and clarity can be considered quality details as they reflect the quality of a statement. In contrast, the number of arguments can be considered a quantity detail because it tells us nothing about the quality of the arguments. The present experiment showed more support for the pre-registered hypothesis regarding the quality details than that regarding the quantity details, and the same pattern emerged in Leal, Vrij, Deeb, and Dabrowna (2023), Mann et al. (2022) and Vrij, Deeb, et al. (2022). When discussing alleged past activities, the variable total details (a quantity measure) typically emerges as a strong veracity indicator: truth tellers report more details than lie tellers (Amado et al., 2016; Gancedo et al., 2021). It thus seems that quantity measures are stronger veracity indicators when discussing past activities than when discussing opinions. We can think of two reasons why this is the case. First, lie tellers are inclined to avoid presenting incriminating evidence. Details about past activities are perhaps easier to check by investigators than details about expressed opinions, and lie tellers are therefore more reluctant to provide details about past activities than details about opinions. Second, it is probably easier to make up many details about opinions than about past activities. Expressing opinions is context free, so if someone has heard or read an opinion, they can easily claim it to be their own. Past activities are embedded in time and location (occurred at a specific moment at a specific location), and such contextual embeddings cannot be easily replaced when making up a story. Lie tellers are therefore motivated to leave contextual embeddings out of their fabricated statements about past activities (Nisin et al., 2022), which contributes to lie tellers reporting fewer details than truth tellers.

Plausibility emerged as the strongest veracity indicator in the present lying about opinions experiment, replicating the findings of several other lying about opinions experiments (Leal, Vrij, Deeb, & Dabrowna, 2023; Mann et al., 2022; Vrij, Deeb, et al., 2022). Researchers are reluctant to examine plausibility, due to its subjective nature: ‘How to define plausibility?’ (Vrij, Deeb, et al., 2021). However, we found good reliability between our coders for plausibility (average measures ICC = .75), which suggests that it is possible to measure it in a reliable way. In addition, when researchers do examine plausibility, it emerges as one of the strongest verbal veracity indicators, not only when people lie about opinions but also when they lie about their alleged activities (Gancedo et al., 2021; Sporer et al., 2021). We think that plausibility deserves more attention from verbal deception researchers than it currently receives.

Although it is always unfortunate if a variable does not emerge as a diagnostic veracity indicator, it is particularly unfortunate for the being scripted variable, because it is a cue to deceit (lie tellers report the cue more than truth tellers). All the other variables we measured were cues to truthfulness (truth tellers report the cue more than lie tellers). Verbal deception research overwhelmingly focuses on cues to truthfulness. All 19 criteria that belong to the Criteria-Based Content Analysis verbal veracity assessment tool are cues to truthfulness (Amado et al., 2016), and one out of eight cues that belong to the Reality Monitoring (RM) tool are cues to truthfulness (Oberlader et al., 2016). The exception is cognitive operations, which was the only RM criterion that did not emerge as a significant veracity indicator in a recent meta-analysis (Gancedo et al., 2021). Besides this, there are difficulties in coding the cognitive operations variable (Vrij, 2008), which we also saw with the being scripted variable. In addition, verifiable details – a key variable in the Verifiability approach (Nahari, 2019) – is a cue to truthfulness, and so are type-token ratio – a key variable in the Reality Interview (Colwell et al., 2013) – and complications – a key variable in Cognitive Credibility Assessment (Vrij, Mann, et al., 2021).

Some cues to deceit have emerged in verbal deception research. Meta-analyses showed that common knowledge details and self-handicapping strategies (Vrij, Palena, et al., 2021) but, particularly, statement–evidence inconsistency and within-statement inconsistency – two key variables in the Strategic Use of Evidence (SUE) tool (Hartwig & Granhag, 2022; Hartwig et al., 2014) – are diagnostic cues to deceit. The problem with the two SUE variables is that the SUE tool can only be used when an investigator possesses independent evidence (witnesses, CCTV footage, fingerprints, etc), which is in many situations not the case.

There are at least two reasons why it is important that researchers continue their search for verbal cues to deceit (Vrij, Fisher, et al., 2023). First, as already mentioned in the introduction, investigators could make their veracity judgements with much more confidence if they examine a mixture of verbal cues to truthfulness and deceit rather than just verbal cues to deceit. Second, it is more natural to people focusing on the presence of a signal (i.e. cue to deceit) than on the absence of a signal (i.e. cue to truthfulness). Since it is more natural to focus on cues to deceit, verbal lie detection may become more popular amongst practitioners if we will be able to advise them what these verbal cues to deceit are. Future research could focus on different cues to deceit, such as the predictability of the answers. Someone who has not thought much about arguments that go against their opinion may come up with arguments that sound obvious or predictable. We thus predict that lie tellers will give more predictable answers than truth tellers.

We made two changes in the Devil’s Advocate protocol compared to Leal et al. (2010) and Mann et al. (2022). First, we asked three eliciting-opinion questions rather than just one. The three questions produced very similar results, which increases confidence that the findings are reliable. Nonetheless, the responses to the three questions showed little overlap; it means that we managed to gather more information regarding why interviewees (allegedly) were in favour of their expressed opinion than we would have obtained with a single question. We therefore recommend continuing using the three eliciting-opinion questions protocol. Second, we simplified the devil’s advocate question. The results contradicted the predictions because truth tellers sounded more (rather than less) plausible, immediate and clear than lie tellers when answering the devil’s advocate question. An explanation would be that truth tellers did not find it difficult to come up with a devil’s advocate answer. This cannot be caused by the topic – lying about actions – because in Mann et al. (2022) participants also discussed protester actions. The most logical conclusion is that the change in wording somehow caused the absence of the effect. Mann et al. (2022, Appendix) asked: *You must have considered your opinion. If you were to look at it from the point of view of an opposer to the actions taken by Matt and Jane, is there anything you can say against their actions? Can you think of any arguments for why Jane and Matt should not take those actions against those individuals*? In the current experiment we simplified it to: *Can you think of any arguments for why Jane and Matt should not take those actions against those individuals?* The main difference between the original question and the rephrased question is that in the current experiment we did not refer to ‘*look at it from the point of view of an opposer to the actions’*. Perhaps this particular phrase is necessary to make people really think about counter-arguments. Indeed, 18 truth tellers and eight lie tellers avoided answering the question by including ‘No’ or ‘Not really’ in their responses, and these answers were rated as more plausible and clearer than the other responses to the devil’s advocate question. The absence of the predicted effect means that the search continues to come up with a good alternative to the original devil’s advocate question.

In the original devil’s advocate protocol (Leal et al., 2010), participants just stated their opinion in the first question, whereas in Mann et al. (2022) and the present experiment participants were asked to elaborate on this and discuss why they had that opinion. Similar to Mann et al. (2022), this question resulted in very similar veracity differences to those resulting from the eliciting-opinion questions. Apparently, lie tellers struggle to sound like truth tellers not only when they express their reasons for having their false opinion (eliciting-opinion questions) but also in expressing the false opinion itself. We therefore recommend maintaining this ‘expressing the opinion’ question at the beginning of the interview because it gives investigators further information about the veracity status of the interviewee.

Three limitations merit discussion. First, the level of activism participants endorsed was rather low. We attempted to raise these levels by presenting participants’ vignettes whereby a protagonist has suffered a clear injustice. We were hoping that these vignettes would stir up empathy/anger in participants so that they would consider it appropriate to take strong actions. The situations the protagonists were in were indeed seen as very unfair and elicited very strong feelings of empathy, yet most participants were not keen on strong actions. If we assume that our participants represent the general public, it suggests that the willingness for protester actions amongst the population is generally low. We think that the devil’s advocate interview tool works better when the participants have more extreme views. The more extreme their views, the less they will consider anti-arguments to be appropriate, so the less they will think about them. The more they reject the anti-arguments, the more they will struggle to express them eloquently in the devil’s advocate question.

Second, similar to Mann et al. (2022), we only audio recorded the interviews. Future research could videotape the interviews so that participants’ nonverbal behaviour could be analysed. Leal et al. (2010) did video-record their participants and found differences in emotional involvement between truth tellers and lie tellers. Truth tellers’ opinion-eliciting answers revealed more emotional involvement than their devil’s advocate answers, whereas no clear differences emerged in lie tellers’ answers to the two types of question. Future research could try to replicate this finding and could examine possible cues to deceit (e.g. being ambivalent or making the impression to try to convince someone). The nonverbal behaviours we have in mind are holistic impressions based on multiple cues rather than the single cue observations (e.g. gaze aversion, fidgeting, micro-expressions of emotions) popular in nonverbal lie detection research (Vrij et al., 2019). Meta-analyses have shown that holistic impressions are better veracity indicators than single-cue analyses (DePaulo & Morris, 2004; Hartwig & Bond, 2014).

Third, similar to Mann et al., (2022), for ethical and practical reasons participants were asked to discuss what actions they considered to be appropriate for others to take rather than actions they may take themselves in the same position. From an ethical standpoint this avoided the event of participants reporting being willing to take strong or illegal actions themselves. From a practical standpoint, since we expected most participants would not be activists themselves we considered they might be more agreeable to other people taking certain actions that carry negative consequences than to taking such risks themselves. For example, someone might have the opinion that Putin should be stopped with lethal force but would probably be more likely to condone someone else taking such action rather than do it themselves. However, we expect participants’ opinions about what action is acceptable for someone else to take would positively correlate with their opinions about what action is acceptable to take themselves, though still this remains an empirical question.

## Appendix

**Table:**

Vignettes used
*Cannabis*
Jane has developed multiple sclerosis and her health and ability to function or hold down her job was suffering badly, until a friend recommended she try using cannabis to help with her pain and symptoms. She was sceptical as she had tried using CBD oil which hadn’t helped at all, but she tried using cannabis and was amazed at how much it helped her. She started using a small amount each evening and was able to resume her job and other activities as well as before her diagnosis. However, the man she bought the cannabis from was apprehended by police and gave Jane’s details as one of his customers. Jane was arrested and, as a result, lost her job whilst awaiting trial. Jane and husband Matt cannot manage the mortgage on their small home just on Matt’s wage and Jane is struggling to get another job, even if she could manage to work without using the cannabis for her symptoms.
*University fees*
Jane, aged 30, decided to pursue her dream job as an English teacher, having become very dissatisfied with her work as an administrative assistant which she had done since leaving school with just GCSEs. Jane was easily capable of doing a degree in English and paid to study for A levels to secure her place at university. She studied hard for these whilst still working and received ‘A’ grades in all three subjects. However, as she was about to start her degree the university fees tripled, and to get into so much debt in order to study further was not possible for her, as she and her husband Matt could not financially manage that and their small home and child. Jane is now 40 and still working as an administrative assistant. Life is hard and she and husband Matt struggle financially, with any spare money they can save going towards their child’s university education.
*Cancer/COVID*
Jane has an aggressive form of cancer. She was told she would qualify for a promising experimental treatment of the disease on the NHS. However, whilst waiting for the treatment the Coronavirus Omicron variant started spreading rapidly and she was informed that she would have to wait for the treatment as it would require a hospital stay and all hospital places were currently full. Jane and her husband, Matt, have heard that the beds are full of unvaccinated patients who have experienced complications with Coronavirus. When Jane was finally called in for her treatment she was informed that her cancer had progressed too far for the experimental treatment to now be a possibility.
*Cost of living crisis*
Jane was very excited to be buying a house finally. She and husband Matt, in their late 30s, had been living in a small damp one-bedroom rented flat for several years whilst saving for a deposit to buy a small home of their own. Jane had been working an evening bar job as well as her full-time day job as a carer in a nursing home, but had to give up her extra job due to exhaustion and the fact that Jane’s elderly mother’s health is now failing, requiring Jane to visit daily and give her substantially more practical help. In addition, Jane suffers from asthma which is exacerbated by the dampness at home. Matt, whose job is based out of town, needs to use their small old car to get to his job. If Jane’s mother lived in a larger house the couple would temporarily move in with her, but she lives in a one-bed cottage. With the dramatic recent increase in fuel prices, and their knock-on effects on the cost-of-living, combined with their reduced income, the couple now have to use their savings just to keep the flat warm and themselves fed. After their car broke down as Matt tried driving home from work they have just learned it needs a new engine which will cost the majority of their remaining savings. Jane had already sold anything of value that they owned and, as further fuel increases are promised, no longer turns on the heating, though the cold makes her more unwell.
